# Anomalous Diffusion of Polyelectrolyte Segments on Supported Charged Lipid Bilayers

**DOI:** 10.3390/e25050796

**Published:** 2023-05-13

**Authors:** Shi Yu, Jianqiao Zhao, Ruizhi Chu, Xiao Li, Guoguang Wu, Xianliang Meng

**Affiliations:** 1 Department of Chemical Engineering, China University of Mining & Technology, Xuzhou 221116, China; jqzhao@cumt.edu.cn (J.Z.); rzchu@cumt.edu.cn (R.C.); lixiao@cumt.edu.cn (X.L.); ggwu@cumt.edu.cn (G.W.);; 2 Key Laboratory of Coal-Based CO_2_ Capture and Geological Storage, China University of Mining & Technology, Xuzhou 221116, China

**Keywords:** subdiffusion, coarse-grained simulation, oxDNA, lipid bilayers

## Abstract

This work provides mesoscale models for the anomalous diffusion of a polymer chain on a heterogeneous surface with rearranging randomly distributed adsorption sites. Both the “bead-spring” model and oxDNA model were simulated on supported lipid bilayer membranes with various molar fractions of charged lipids, using Brownian dynamics method. Our simulation results demonstrate that “bead-spring” chains exhibit sub-diffusion on charged lipid bilayers which agrees with previous experimental observations for short-time dynamics of DNA segments on membranes. In addition, the non-Gaussian diffusive behaviors of DNA segments have not been observed in our simulations. However, a simulated 17 base pairs double stranded DNA, using oxDNA model, performs normal diffusion on supported cationic lipid bilayers. Due to the number of positively charged lipids attracted by short DNA is small, the energy landscape that the short DNA experiences during diffusion is not as heterogeneous as that experienced by long DNA chains, which results in normal diffusion rather than sub-diffusion for short DNA.

## 1. Introduction

The adsorption of polymers onto a planar surface has been studied for several decades [1,2,3,4,5,6,7,8], not only because it is of long-standing interest in polymer physics, but also because it is important in biological interactions [9,10,11]. While early researches focus more on polymers adsorbed on flat rigid surfaces [1,2,7], polymer adsorption on heterogeneous surface attracts more interest [4,5,8,12,13], as many naturally occurring surfaces are energetically inhomogeneous [4]. Chang et al. [14] observed that λ-DNA molecules exhibit sub-diffusion on supported cationic lipid membranes by fluorescence microscopy. Since adsorbed polyeletrolytes on the lipid membranes might introduce rearrangements of the lipid molecules within the membrane [15], Chang [14] also observed a slower DNA conformational relaxation. Moreover, Chang and his/her co-workers [14] found that the exponent characterizing the sub-diffusion (i.e., α in Equation (1) below) approximately equals to 0.38 at a short time scale. Note that the ensemble-averaged, 2D mean-squared displacement (*MSD*) of DNA can be quantified by Equation (1), where *D* and *t* are diffusivity and time lag, respectively.

(1) $$MSD=4Dt^{\alpha }$$

On the other hand, Javer et al. [16,17] demonstrated that loci on bacterial chromosome also perform sub-diffusion with a very similar exponent α (≈0.4). This similarity between the motions of DNA segments adsorbed on lipid membranes and that of loci in bacterial cell seems to suggest that the sub-diffusion of DNA segments in both cases originates from random traps. S. Burov and E. Barkai [18] investigated the occupation time statistics of particles in quenched trap, and diffusive behaviors of polymers in quenched disordered media have been studied by various simulation techniques [19,20,21]. However, to elucidate how the rearranging energy traps (e.g., diffusive cationic lipid molecules within lipid membranes) affect the motions of polymer, a model with appropriate resolution which incorporates the degrees of freedom of the rearranging energy traps is required. This manuscript aims to develop a coarse-grained model to simulate the motions of DNA/polyelectrolytes on lipid bilayer membranes with rearranging traps.

As biological membranes which may contain many different types of lipids are essential components of every cell, various lipid membranes models at different scales have been developed [22,23]. As many cellular processes may take place over large time and length scales, a bunch of coarse-grained lipid models have been proposed recently, such as Shinoda/Devane/Klein (SDK) model [24,25], Martini model [26], and ELBA force field [27], etc. Such coarse-grained lipid models use 3∼4:1 mapping (i.e., 3∼4 atoms are represented by a single bead) to reduce the degrees of freedom of the simulation system. To further speed up the simulation of lipid membranes, Cooke et al. [28,29] developed a solvent-free generic mesoscopic model of lipid membranes which uses only three connected beads (one hydrophilic “head” bead and two hydrophobic “tail” beads) to simulate a single lipid molecule. In addition, the physical properties of lipid membranes (e.g., bending stiffness or lateral diffusivity of lipid molecule within the membrane) can be tuned by varying a single parameter of this generic model [29]. The dynamics of lipid membranes with supports were investigated using this Cooke’s model by Hoopes et al. [30]. Moreover, R. Sknepnek et al. [31] observed the solidification of lipid membranes by assigning charges to the “head” beads with Cooke’s model. Thus, Cooke’s model with charged “head” beads can be employed to simulate the 2D heterogeneous surface with rearranging energy traps as a platform on which DNA molecules diffuse, as the charged lipid molecules can perform lateral diffusion within bilayer membranes in fluid phase.

Since deoxyribonucleic acid (DNA) plays a crucial role in biological reactions, many coarse-grained models [32,33,34,35,36,37,38,39,40,41,42,43] of DNA have been developed to sample the conformation of large structures of DNA. In addition, the interactions between cationic lipids and DNA have been studied using both simulations and analytical methods [44,45]. It is worth mentioning that coarse-grained oxDNA model proposed by T. E. Ouldridge et al. [39,40,41,42] has similar resolution as Cooke’s generic lipid model [29], and is available as a module for the popular LAMMPS simulation package [46]. Hence, a combination of oxDNA model and Cooke’s lipid model can be a faster alternative of Martini model [38] (4:1 mapping), and can easily be implemented in LAMMPS package. Note that the self-assembly, structural, mechanical and thermodynamic properties of DNA can be captured by oxDNA model [42]. Another advantage of oxDNA model is that ions and solvent molecules in oxDNA model are considered implicitly, which is compatible with Cooke’s lipid model.

As we seek to investigate to what extent the sub-diffusion of DNA/polyelectrolytes segments can be attributed to the random rearranging traps generated by diffusing cationic lipid molecules, this manuscript focuses on the electrostatic interaction between negatively charged DNA chain and positively charged lipid molecules by combining Cooke’s lipid model and DNA models. Both a negatively charged “bead-spring” model and an oxDNA model were used to simulate DNA or polyelectrolytes on lipid bilayer membranes (Cooke’s generic lipid model) in this manuscript. To avoid the interactions among different DNA chains, all simulations were carried out in diluted systems. The structures of both dsDNA and ssDNA are largely affected by the salt concentrations [47] and biological crowders [48], since the electrostatic potential plays an important role in DNA dynamics. As both Cooke’s lipid model and DNA models are solvent free, the electrostatic interactions in all our simulations were modeled by Debye–Hükel theory, which is merely an approximate theory. Recently, the structures and dynamics of DNA in cell-like environments attract more and more interests [49,50,51]. To tackle such problems, finer DNA models with more details about local interactions are required.

In addition, the displacement distributions of DNA beads on charged lipid bilayers were examined in this manuscript to check whether the diffusive motions of those DNA beads are non-Gaussian.

## 2. Simulation Model and Method

### 2.1. Lipid Model

In this manuscript, lipid molecules were modeled by a “head” bead followed by two “tail” beads, which was proposed by Cooke et al. [28,29]. Those three beads are connected by two FENE bonds:

(2) $$U_{bond}\left(r\right)=-\frac{1}{2}k_{bond}r_{\infty }^{2}log\left[1-{\left(\frac{r}{r_{\infty }}\right)}^{2}\right]$$

where *r* is the bond length, $k_{bond}$ =30 $\epsilon /\sigma^{2}$ is bonding force constant, and $r_{\infty }$ = 1.5 σ is divergence length. Note that ϵ is the unit of energy of this model, and σ=1 nm is the unit of length [30]. Each lipid molecule is straightened by a harmonic potential between the “head” bead and the second “tail” bead at the other end of the molecule, described as follows.

(3) $$U_{bend}\left(r\right)=\frac{1}{2}k_{bend}{\left(r-4\sigma \right)}^{2}$$

where the bending stiffness $k_{bend}=10\epsilon /\sigma^{2}$. The repulsive force between each pair of beads is represented by a Weeks–Chandler–Andersen potential [29]

(4) $$U_{rep}\left(r;b\right)=\left\{\begin{array}{c} 4\epsilon \left[{\left(\frac{b}{r}\right)}^{12}-{\left(\frac{b}{r}\right)}^{6}+\frac{1}{4}\right],\;r\leq r_{c}\\ 0,\;\;\;\;\;\;r>r_{c} \end{array}\right.$$

where $r_{c}=2^{1/6}b$, $b_{head,head}=b_{head,tail}=0.95\sigma$ and $b_{tail,tail}=\sigma$. All “tail” beads attract each other based on the equation below. This is to ensure that lipids can self-assemble into bilayers at appropriate temperatures.

(5) $$U_{attr}\left(r\right)=\left\{\begin{array}{c} -\epsilon ,\;\;\;\;\;\;\;\;\;r<r_{c}\\ -\epsilon cos^{2}\frac{\pi (r-r_{c})}{2w_{c}},\;\;r_{c}\leq r\leq r_{c}+w_{c}\\ 0,\;\;\;\;\;\;\;r>r_{c}+w_{c} \end{array}\right.$$

where $w_{c}$ is the tuning parameter in Cooke’s model [29]. In addition, in this paper, $w_{c}$ was fixed to 1.3 σ for simplicity.

The electrostatic interactions were approximated by the Debye–Hückel potential as described below:

(6) $$U_{elec}=\epsilon l_{B}q_{i}q_{j}\frac{e^{-\kappa r_{ij}}}{r_{ij}},\;r_{ij}<2.5r_{c}$$

where κ is the inverse screening length (inverse Debye length); $r_{ij}$ is the distance between the *i*th bead and the *j*th bead; $q_{i}$ and $q_{j}$ are the number of charges of *i*th and *j*th particles, respectively. In this work, the inverse Debye length κ was varied from 0.333 to 3.333, which corresponds to the range of aqueous salt concentrations from 10 mM to 1 M at 25 ${}^{{}^{\circ}}$C. The $l_{B}$ in equation above is the Bjerrum length, as defined below:

(7) $$l_{B}=\frac{e^{2}}{4\pi \varepsilon_{0}\varepsilon k_{B}T}$$

where $\varepsilon_{0}$ is the permittivity of vacuum, and ε is the dielectric constant. Since the value of $l_{B}$ in water at 25 ${}^{{}^{\circ}}$C is 7.1 Å, the $l_{B}$ in this work was fixed to 0.71 σ.

### 2.2. Lipid Bilayers with Supports

Lipid bilayer membranes with supports are an important platform for physical measurements of fluid bilayers [14,30]. Hoopes et al. [30] used Cooke’s lipid model and a simple particle-based realization for a flat support to investigate the lipids adsorption onto the surface. In addition, Hoopes [30] proposed an analytic expression for the asymptotic interaction potential between lipids and flat surface which is described as follows.

(8) $$\frac{U_{asymp}\left(r\right)}{\epsilon }=\frac{16\pi }{\sqrt{3}}\left\{\frac{1}{10}{\left(\frac{\sigma }{r}\right)}^{10}-\frac{1}{4}{\left(\frac{\sigma }{r}\right)}^{4}\right\}$$

Since all the simulations of lipids with supports in this manuscript were implemented in LAMMPS package, the adjusted wall/lj1043 potential (as shown in equation below) which is a build-in function of LAMMPS software was adopted instead of the asymptotic interaction potential proposed by Hoopes et al. [30] to model the interaction energy between the lipid head group and the flat surface.

(9) $$\frac{U_{wall}}{\epsilon }=3.1454\pi \left[\frac{2}{5}{\left(\frac{\sigma }{r}\right)}^{10}-{\left(\frac{\sigma }{r}\right)}^{4}-\frac{\sqrt{2}\sigma^{3}}{3{\left(r+(0.61/\sqrt{2})\sigma \right)}^{3}}\right],\;r<2.5\sigma$$

**Figure 1:**
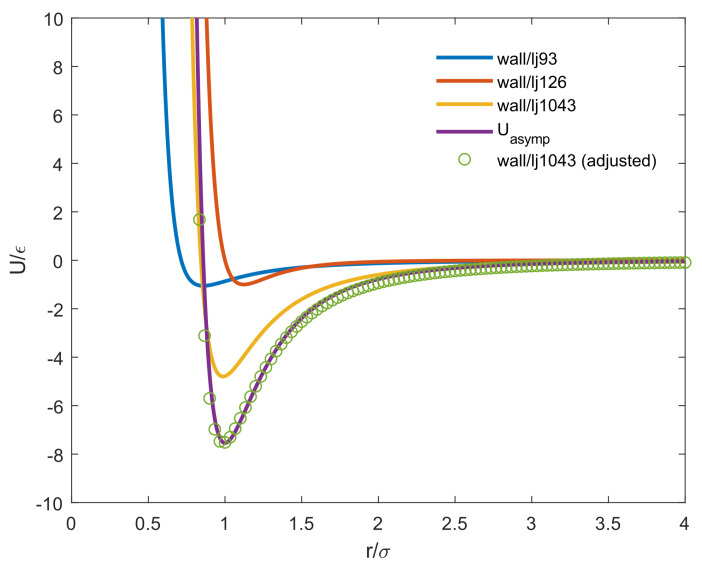
Different types of interaction energy as a function of vertical position (in *Z* direction) for a lipid head group placed over the flat surface.

As demonstrated in Figure 1, the adjusted wall/lj1043 potential (Equation (9)) and the asymptotic interaction potential (Equation (8)) coincide with the wide range of distance between particle and flat surface.

### 2.3. DNA Models

**Figure 2:**
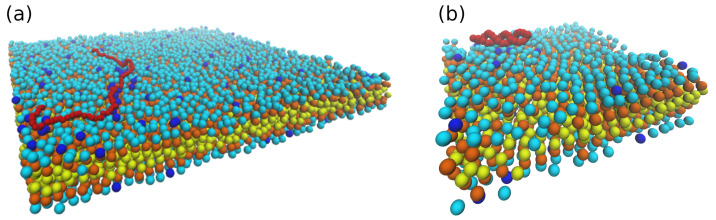
(**a**) Snapshot of 100 beads DNA (“bead-spring” model, depicted as red beads) on weakly charged lipid bilayer with supports (“head” beads and two “tail” beads are depicted as cyan, orange, and yellow beads, respectively; κ=0.333). (**b**) 17 bp dsDNA (“oxDNA” model, red beads) on charged lipid bilayer (inverse Debye length κ=0.333).

As demonstrated in Figure 2, both a simple “bead-spring” model and oxDNA model were adopted to simulate the diffusive motions of DNA segments on lipid bilayer membranes with supports in this work. For “bead-spring” model, consecutive beads are connected by “finitely extensible nonlinear elastic” (FENE) bonds [52,53], and the bonding potential is given by Equation (10).

(10) $$U_{FENE}=-\frac{1}{2}KR_{0}^{2}ln\left[1-{\left(\frac{r}{R_{0}}\right)}^{2}\right]$$

where $R_{0}$ = 0.8 σ; *K* = 30.0 $\epsilon /\sigma^{2}$. The electrostatic interactions between each pair of negatively charged DNA beads and that between DNA beads and positively charged lipid “head” beads were also modeled by Debye–Hückel potential (Equation (6)). Note that the number of charges on each DNA bead ($q_{i}$) was set to −2.94. In addition, the excluded volume potential between a DNA bead and a lipid bead was modeled by Weeks–Chandler–Anderson potential (Equation (4)), where $b_{DNA,lipid}$ = 1.0 σ.

For oxDNA model, only two different interactions were considered in our simulations. One is the electrostatic attraction between cationic “head” bead of lipids and oxDNA beads. In addition, the other is the excluded volume interaction between each pair of lipid bead and oxDNA bead. Again, the electrostatic interaction was described by Debye–Hückel potential as shown in Equation (6), and the number of charges on oxDNA beads was set to −1.0. In addition, the excluded volume potential between Cooke’s model lipid beads and oxDNA beads were modeled by Weeks–Chandler–Anderson potential, just like the “bead-spring” model of DNA as discussed above. However, for the implementation of oxDNA model in LAMMPS software, 1.0 length unit (σ) = 0.85 Å [41]. Therefore, to combine Cooke’s lipid model and oxDNA model in LAMMPS software, the length unit of Cooke’s lipid force field was converted to the length unit of oxDNA model before each run. For direct comparison between “bead-spring” model of DNA and oxDNA model, the unit length of the Brownian dynamics simulation results of oxDNA-lipids complexes were converted back to the length unit of Cooke’s model (i.e., length unit σ=1 nm).

### 2.4. Simulation Method

Cooke’s lipid model [29] was employed for all our Brownian dynamics simulations performed in this work, using LAMMPS package [46]. For simulations of lipid bilayer membranes in bulk as well as on flat surface, 4172 lipid molecules were positioned randomly in a 50×50×40$\sigma^{3}$ simulation box initially. The simulation system was first equilibrated by performing a $5\times 10^{6}$ time steps simulation with a time step δt=0.001. Then, another $1\times 10^{7}$ time steps simulation was performed to track the lipids motions in our simulation systems with the same time step δt as in the equilibration stage. For our simulations of lipid bilayer membranes with supports, all lipid molecules were pushed down onto the flat reflecting surface at z=0 in the equilibration stage to generate lipid bilayer membranes on supporting surface, by applying external forces on all lipid beads. After the equilibration, the external forces exerted on lipid beads were removed to obtain the correct dynamics of lipid molecules. In addition, for simulations of lipid membranes with supports, the adjusted wall/lj1043 potential (Equation (9)) was applied between lipid “head” bead and the bottom wall of the simulation box at z=0, while in all other cases the top wall at z=40 and the bottom wall at z=0 act as reflecting walls when they interact with lipid beads. To investigate how the molar fraction of charged lipids (Φ) affects the diffusive behaviors of lipids, Φ was varied from 0.0 to 0.1 by randomly assign 1.0 positive charge to different numbers of “head” beads of the system.

The simulations of “bead-spring” chains diffusion on lipid bilayers were performed by placing a single polymer chain in the simulation box with supported partially charged lipid bilayers as discussed above. The effects of ionic strength of the solution on DNA segments diffusion were examined by varying the inverse screening/Debye length κ from 0.333 to 3.333. The simulation system which is consist of a single “bead-spring” chain and 4172 lipid molecules was first equilibrated by $5\times 10^{6}$ time steps (δt=0.001). Then, like the simulation of lipid membranes, $1\times 10^{7}$ time steps simulation of “bead-spring” chain on lipid bilayers was carried out to study the diffusion of DNA segments on positively charged bilayer membranes.

To combine the oxDNA model and Cooke’s lipid model, a 17 base pairs double-stranded DNA (base pair sequence (5${}^{\prime }$-3${}^{\prime }$): TGAACAGACGCTGCTGC) was introduced to a 20×20×20 $\sigma^{3}$ (σ here is the unit length of Cooke’s model) simulation box which contains 668 lipids. Since many potentials of the oxDNA model implementation in LAMMPS are temperature dependent, the temperature of the oxDNA in reduced LJ-units (i.e., $k_{B}T/\epsilon$) has to be set to 0.1 to ensure that the predicted persistent length and melting temperature of oxDNA model agree with experimental values. However, this specific implementation of oxDNA model obstructs the observations of diffusive behaviors of oxDNA, for it is necessary to assign different temperatures to oxDNA molecule and to those lipid molecules. This is due to the charged lipid bilayers at T=0.1 (in reduced LJ-units) is in gel phase [29], where the lateral diffusion of lipids ceases. In addition, the energy landscape generated by such lipid bilayers at low temperature is consist of quenched energy traps rather than rearranging traps that we aim to investigate. Therefore, the oxDNA temperature was fixed to 0.1, whereas lipids temperature was varied from 0.8 to 1.0 in our Brownian dynamics simulations of oxDNA-lipid bilayer complexes. In addition, for all those oxDNA-lipids complexes simulations, $1\times 10^{8}$ time steps simulations were carried out with a time step δt=0.001 after a $5\times 10^{6}$ time steps equilibration.

## 3. Results and Discussion

### 3.1. Simulations of Charged Lipid Bilayer Membranes

**Figure 3:**
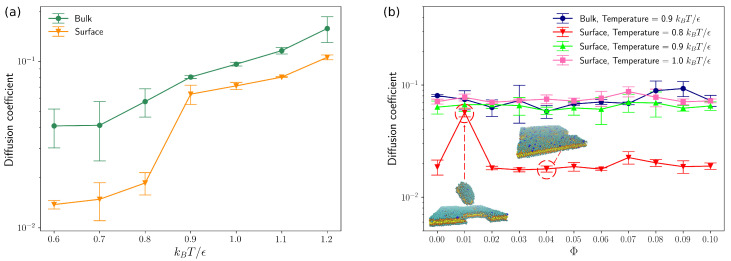
(**a**) Diffusion coeffcients of lipids in bulk and on surface as a function of temperature. (**b**) Diffusion coeffcients of lipids in bulk and on surface as a function of molar fractions (Φ) of charged lipids. (The error bars are standard deviations and inverse Debye length κ=0.333).

As described above, we use Cooke’s lipid model [29] to simulate the neutral lipid bilayer membranes in bulk as well as neutral bilayers on surface at different temperatures. The average diffusion coefficients of all lipid beads in the simulation box were obtained and plotted against system temperature in reduced LJ-units ($k_{B}T/\epsilon$), as demonstrated in Figure 3a. As temperature increases, the average diffusion coefficient of all lipids also increases. In addition, as can be seen in Figure 3a, a phase transition from “gel” to “fluid” phase takes place between 0.7 and 0.8 $k_{B}T/\epsilon$ for lipid bilayers in bulk, which agrees with Cooke’s simulation results [29]. A similar transition takes place around T=0.8 for lipid bilayer membranes on surface, which indicates that the attraction force between “head” beads on lipids and the flat surface (Equation (9)) can suppress the diffusive motions of lipids, as most of lipids condense closed to the flat surface at z=0.

The lipid bilayer membranes with various molar fractions (Φ) of charged lipids were also studied by Brownian dynamics in bulk and on surface. As will be discussed below, the molar fraction (Φ) of charged lipids was varied from 0.0 to 0.1, which allows us to compare our simulation results of DNA-lipid bilayer complexes directly to previous experimental measurements carried out by Chang et al. [14]. As shown in Figure 3b, the molar fraction of charged lipids has negligible effects on average diffusion coefficients of lipid molecules, as long as Φ≤0.1. Sknepnek et al. [31] performed a series of molecular dynamics simulations of charged lipids by extending Cooke’s lipid model [29] and observed solidification of lipid bilayers when each “head” bead was assigned different number of charges. This “solidification” of lipid bilayers was not found in our simulation results for the charged densities of our systems are much lower compared to that of Sknepnek’s work [31]. As can be seen from Figure 3, the average diffusivity of lipids on surface increases significantly as temperature *T* increases from 0.8 to 0.9, which is similar to the simulation results of neutral lipids on surface (Figure 3a). One outlier occurs at T=0.8 for Φ=0.01, resulting from a large diffusing fragment of membranes as demonstrated in Figure 3b. For all other cases, most lipids stay in the lipid bilayers closed to the bottom surface of the simulation box. As lipids in membranes at higher temperatures (T≥0.9) can perform lateral diffusion in fluid phase, the lipid bilayer membranes with supports that we constructed here could be a good platform for us to investigate the effects of rearranging energy traps on polyelectrolytes diffusion.

### 3.2. “Bead-Spring” Chain on Lipid Bilayer Membranes

**Figure 4:**
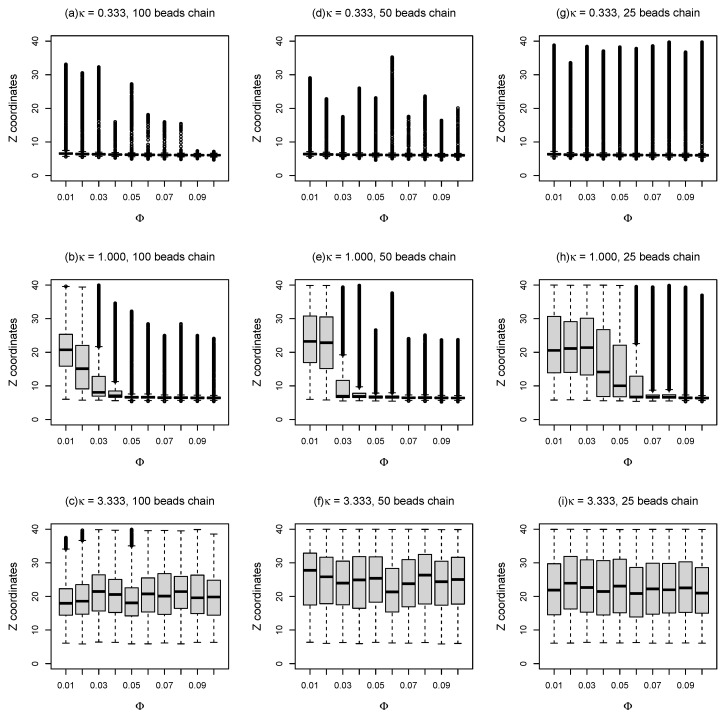
*Z* coordinates of the DNA bead in the middle of the chain as a function of molar fractions (Φ) of charged lipids for different κ and different chain lengths.

In order to gain some insight into the sub-diffusion of dsDNA on weakly charged lipid bilayer membranes with supports, as discussed above, “bead-spring” model of DNA was employed to simulate double-stranded DNA of different lengths on partially charged lipid bilayers using Brownian dynamics simulations, as shown in Figure 2a. Three different types of “bead-spring” chains (i.e., 100, 50, and 25 “beads” chains) were simulated with lipid bilayers. Note that a direct comparison between this mesoscopic model (i.e., “bead-spring” chains on Cooke’s lipid bilayers) and the real atomistic scale simulations is not precisely defined. However, since this work partly focused on the effects of rearranging traps on diffusive motions of polyelectrolytes, this mesoscale model which incorporates cationic lipids as diffusing energy traps and polyelectrolytes with similar resolution might suffice. To determine whether the “bead-spring” chains adsorbed onto charged lipid bilayers, the box plots of coordinates distributions of the beads in the middle of the chain in *Z* direction during the simulations are plotted against the molar fraction of charged lipids (Φ), as demonstrated in Figure 4. The outliers in Figure 4 are labeled by black circles. As expected, for small inverse Debye length (κ=0.333), the bead in the middle of the chain remains on the surface of lipid bilayer membranes on flat surface during the entire simulations ($1\times 10^{7}$ time steps with δt=0.001). As charge density of the lipid bilayer increases, the number of outliers reduces as shown in Figure 4a. Even for short “bead-spring” chain (25 beads) as shown in Figure 4g, the bead in the middle of the chain is almost confined on the surface of lipid membranes, although the attraction force between short chain and lipid bilayers is significantly weaker. As demonstrated in Figure 4b, the “bead-spring” chain begins to detach from lipid bilayer surface when κ is set to 1.0 for small molar fraction of charged lipids (Φ≤0.03). Note that the “detachment” here means that the average *Z* coordinates of the middle bead is larger than the sum of the thickness of lipid bilayer (≈6) and the triple Debye lengths (3×1.0) in this case (as shown in Figure 4b). This detachment is easier to take place for shorter chains (25 beads chain), as shown in Figure 4h.

For a large inverse Debye length (κ=3.333), as shown in Figure 4c,f,i, the electrostatic attractions between negatively charged “bead-spring” chain and positively charged lipids are screened out. As a result, the middle beads of polymer chains tend to perform random walks so that the mean *Z* coordinates of the middle beads are around z=20 during the simulations, as the lipid bilayers act like the top reflecting walls positioned at z=40 in this case.

**Figure 5:**
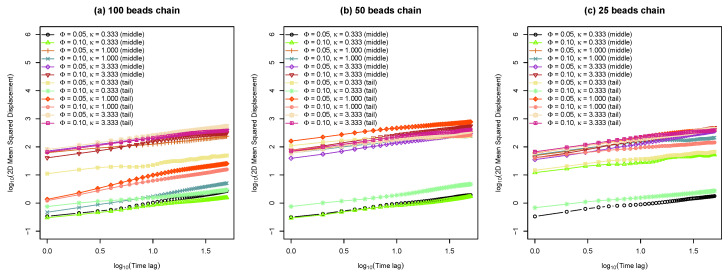
(**a**) Average two-dimensional (xy plane) mean squared displacement of middle and tail beads on a 100 beads chain versus time lag for various molar fractions of charged lipids (Φ) with different inverse Debye length κ. (**b**) Average 2D MSD of middle and tail beads on a 50 beads chain as a function of time lag for different Φ and κ. (**c**) Average 2D MSD of middle and tail beads on a 25 beads chain as a function of time lag with different Φ and κ.

In order to quantify the diffusive motions of charged “bead-spring” polymer chains on lipid bilayers, the 2D mean squared displacements of both the bead in the middle of the chain and that at the end of the chain were obtained. In addition, several example tracks of 2D MSD in xy plane are plotted against the time lag in Figure 5. The 2D mean squared displacement can be calculated by equation below.

(11) $$MSD_{2D}\left(\Delta t\right)=\langle {\left[x(t+\Delta t)-x(t)\right]}^{2}+{\left[y(t+\Delta t)-y(t)\right]}^{2}\rangle$$

where angle brackets 〈〉 represent ensemble average; x(t) and y(t) are the positions of the beads in *x* and *y* directions at time *t*; and Δt is the time lag. As can be seen in Figure 5, many example MSD tracks are very closed to straight line for log scale axises. Moreover, the slopes of those MSD tracks are much smaller than 1.0 which indicates that the DNA segments exhibit sub-diffusion using this simple “bead-spring” polymer model in combination with Cooke’s mesoscopic lipid model. We also observed that the motions of polymer beads are slowed down dramatically by reducing inverse Debye length κ, in other words, by reducing salt concentrations. This observation agrees with the simulation results that “bead-spring” chains are confined very closed to the surface of the lipid bilayer membranes which is shown in Figure 4, as discussed above. In addition, the tail beads on polymer chains move faster compared to the middle beads, because the tail beads are less constrained by the “bead-spring” chain.

**Figure 6:**
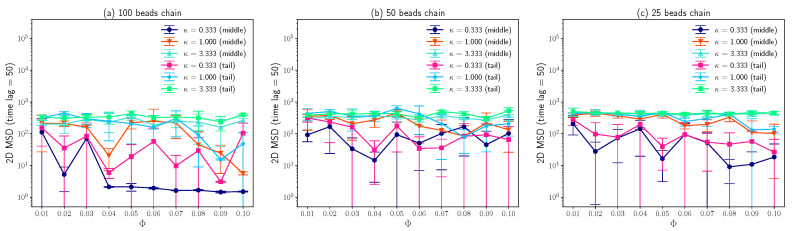
(**a**) Average 2D MSD of middle and tail beads on a 100 beads chain for time lag Δt=50 as a function of molar fraction of charged lipids Φ for different inverse Debye length κ. (**b**) Average 2D MSD of middle and tail beads on a 50 beads chain for Δt=50 vs. Φ for different κ. (**c**) Average 2D MSD of middle and tail beads on a 25 beads chain for Δt=50 vs. Φ for different κ. The error bars are standard deviations.

As demonstrated in Figure 6, the 2D mean squared displacements of both the middle beads and tail beads over Δt=50 time lag are plotted as a function of molar fraction of charged lipids Φ. Due to the strong attraction between 100 beads chain and lipid bilayers for small inverse Debye length (κ=0.333), the MSD for time lag Δt=50 is about two orders of magnitude smaller than that for large κ (3.333), as long as molar fraction of charged lipids Φ is larger than 0.03. For a large inverse Debye length (κ=3.333), the mean squared displacements are about 300 for different molar fractions of charged lipids, because the electrostatic interactions between lipids and DNA have been screened out. In addition, these results agree with the observations about *Z* coordinates distributions of DNA beads which is shown in Figure 4. For other cases, the large error bars which represent the standard deviations of three measurements imply that the diffusive motions of “bead-spring” chains on lipid bilayer membranes are highly random. Since the positively charged lipids performing lateral diffusion can condense around negatively charged “bead-spring” chains and generate energy traps with random depths, the polymer chain adsorption onto lipid membranes and polymer desorption from lipid membranes are also random, which agrees with the results that the *Z* coordinates distributions have lots of outliers shown in Figure 4. As “bead-spring” chains away from lipid bilayer membranes can diffuse much faster than that confined by charged lipid bilayers, the 2D MSD of “bead-spring” chains is dominated by motions of chains away from lipid bilayers which highly depends on the random adsorption/desorption cycles of “bead-spring” chains. This also explains the large standard deviations of 2D MSD measurements.

**Figure 7:**
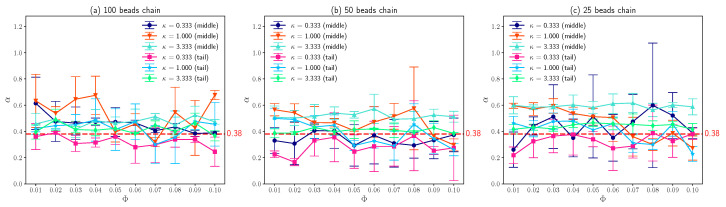
(**a**) The exponent α of middle beads and tail beads on a 100 beads polymer chain which can characterize the sub-diffusion as a function of molar fraction of charged lipids Φ with different inverse Debye length κ. (**b**) The exponent α of middle beads and tail beads on a 50 beads chain as a function of Φ with different κ. (**c**) The exponent α of middle beads and tail beads on a 25 beads chain as a function of Φ with different κ. The error bars are standard deviations.

The most interesting results obtained in this work is that the fitted exponent α based on Equation (1) approximately equals to 0.38 as shown in Figure 7, which agrees well with experimental observations [14]. Assume that the simulation time is long enough which allows the DNA with finite size to pass through the simulation box many times, it is not difficult to conclude that the fitted exponent α in this case equals to one based on the symmetry of the system, since periodic boundary conditions were applied in *x* and *y* directions. Or, in other words, DNA chains perform normal diffusion for large time scale. Actually, Chang et al. [14] did observe this transition of α from 0.38 to 1.0. Hence, for large time scale, the effects of positively charged lipids slowing down the diffusion of DNA segments can be absorbed into the apparent diffusion coefficient, as described by equation below [54].

(12) $$D_{app}=\frac{D_{0}}{\langle e^{\frac{V}{k_{B}T}}\rangle \langle e^{-\frac{V}{k_{B}T}}\rangle }$$

where $D_{0}$ is the diffusion coefficient without external potential landscape; *V* is the external periodic potential. On the other hand, for small time scale (not that small), the potential landscape that the DNA segment experiences is heterogeneous, as the number of the oppositely charged lipids condense around DNA is random. As a result, for short time scale, DNA segments exhibit sub-diffusion. As shown in Figure 7, although the error bars (standard deviations of three simulations) are large for different molar fractions of charged lipids with different κ, the average α is very closed to 0.38. Note that similar phenomenon also takes place for loci in bacterial cell [16,17], where the average value of fitted α is about 0.4 and the distribution of α is wide. Since the diffusive motion of DNA on charged lipid bilayers is a combination of two random processes, that is, the DNA bead motions due to thermal fluctuations and the interaction between DNA segments and a random number of charged lipids, the wide distribution of fitted exponent α is understandable.

**Figure 8:**
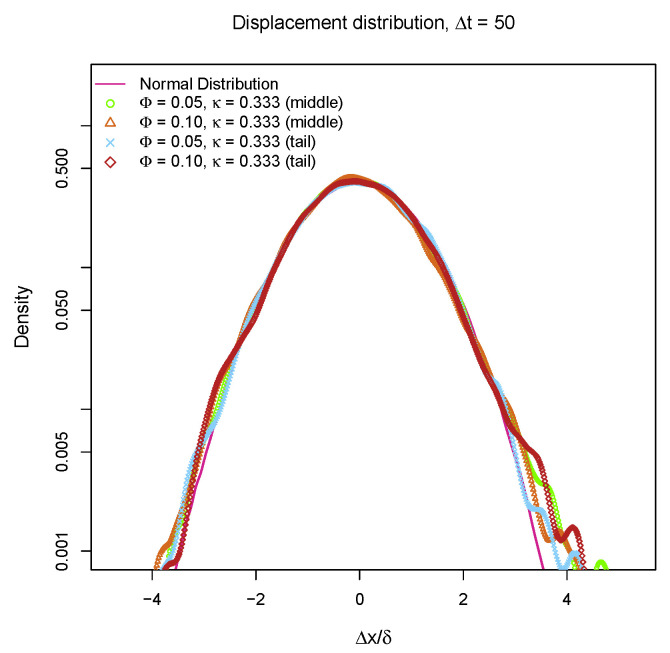
1D displacement (Δx/δ) distributions of middle bead and tail bead on a 100 beads chain over Δt=50 with different molar fraction of charged lipids Φ and different inverse Debye length κ. δ is the standard deviation of Δx.

Many particles in heterogeneous environment exhibit non-Gaussian behaviors which has been thoroughly investigated [55,56,57]. In order to determine whether the “bead-spring” chains also perform non-Gaussian diffusion on partially charged lipid bilayers, the distributions of one-dimensional displacements over Δt=50 are plotted in Figure 8. The 1D displacement distributions of the beads in the middle and at the end of “bead-spring” chain coincide with normal distribution which is depicted as a magenta line. These results indicate that the diffusive motions of “bead-spring” chains can still be described by Gaussian statistics. Although the partially charged lipid bilayer membranes with rearranging traps result in sub-diffusion of “bead-spring” chain which is similar to the sub-diffusion of loci in cytoplasm [55], the viscoelasticity which exists in cytoplasm is missing for weakly charged lipid bilayers, as those positively charged lipids can perform lateral diffusion in fluid phase, especially considering the fact that the diffusivity of lipids is not reduced by introducing charges as shown in Figure 3b. So, the model proposed in this work might be able to explain the sub-diffusion of DNA segments on lipid membranes [14], but it cannot fully describe the anomalous diffusion of particles in cytoplasm [55,56], where the viscoelasticity of the cytoplasm plays an important role. Another major difference between sub-diffusion of DNA on weakly charged lipid bilayers and sub-diffusion of particles in cytoplasm is the metabolic activities are critical for cytoplasmic diffusion [58].

### 3.3. oxDNA Model

Although coarse-grained force fields such as Martini FF [38] can be applied to DNA–lipid bilayer membranes systems, such 4:1 mapping coarse-grained model still contains a very large number of degrees of freedom compared to mesoscopic models of lipids and DNA. Theoretically, the combination of oxDNA model and Cooke’s lipid model can speed up the simulation of DNA on lipid bilayers dramatically. However, it is still challenging to combine these two mesoscopic models and compare this combination of two mesoscale models to atomistic scale simulations directly. The major difficulty is that the implementation of the oxDNA model in LAMMPS package can only provide precise structural and dynamical information of DNA molecules when the temperature of simulation system is fixed to T=0.1, which is not compatible with Cooke’s simple lipid model [29]. Due to this limitation, different temperatures were assigned to the 17 base pairs oxDNA model and lipids with different Φ. As a result, precise dynamics of oxDNA on lipid membranes can hardly be obtained using this simple mesoscopic models combination. Nevertheless, it is still possible for this combination of oxDNA and Cooke’s lipid model to provide meaningful structural information of the equilibrated state of the oxDNA–lipid complexes, as long as lipid membranes are not disrupted strongly by a oxDNA chain.

**Figure 9:**
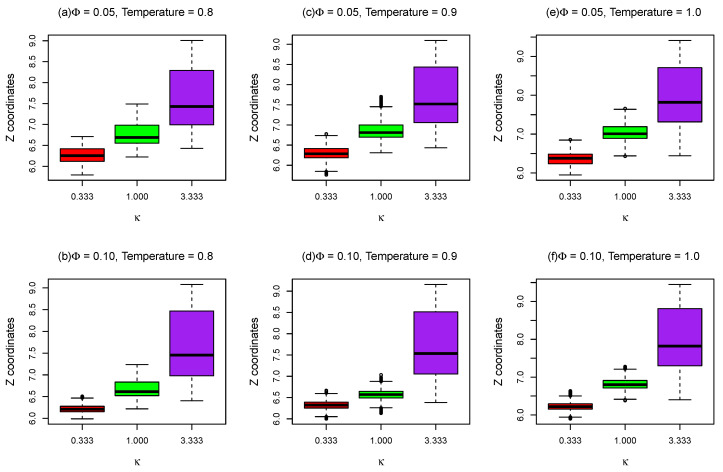
*Z* coordinates of the DNA bead (oxDNA model) in the middle of the chain as a function of κ for different temperatures and different molar fractions (Φ) of charged lipids.

As shown in Figure 9, the positions distributions of the beads in the middle of the 17 base pairs oxDNA chains in *z* direction as a function of inverse Debye length κ are plotted with different Φ and at different temperatures of lipid bilayer membranes. For a small κ (0.333), the oxDNA adsorbs on the lipid bilayer membrane surface during the entire simulation ($1\times 10^{8}$ time steps). As molar fraction of charged lipids increases from 0.05 to 0.1, the *Z* coordinates distributions of oxDNA beads during the simulations become narrower, as demonstrated in Figure 9, since the electrostatic attraction between oxDNA and lipid bilayers with Φ=0.1 is stronger. Due to the electrostatic interactions between oxDNA and lipid bilayers are screened out for large inverse Debye length κ, the *Z* coordinates distributions of oxDNA bead become wider and the average positions of oxDNA middle bead in *z* direction become larger, as shown in Figure 9. Since only electrostatic interaction and excluded volume potential between oxDNA and lipid bilayer membranes were considered in this mesoscopic model, the simulation results presented here cannot be compared to atomistic simulation results or finer coarse-grained simulation results [38] directly. However, if the implementation of oxDNA model in LAMMPS has been improved which allows us to set a uniform temperature to our simulation system, the interaction between oxDNA and lipids can be further tuned to ensure that the potential mean force (PMF) of binding of oxDNA to Cooke’s lipid bilayers agrees with previous atomistic simulation results [38].

**Figure 10:**
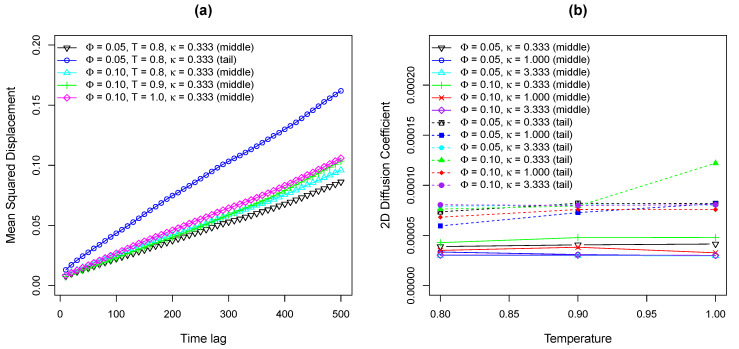
(**a**) Example 2D MSD tracks of middle bead and tail bead on a 17-bp double-stranded oxDNA model versus time lag with various Φ and κ. (**b**) Fitted 2D diffusion coefficients of middle bead and tail bead on a 17-bp oxDNA chain as a function of temperature of lipid bilayer membranes with different Φ and κ.

As shown in Figure 10a, several example 2D MSD tracks of oxDNA middle and tail beads are plotted against time lag Δt with two different molar fractions of charged lipids at different temperatures of lipid molecules. The major difference between oxDNA and “bead-spring” model is that the 17 base pairs oxDNA double strands exhibit normal diffusion on lipid bilayer membranes based on the fact that the MSD tracks of oxDNA are straight lines in Figure 10a. The reason that the 17 base pairs oxDNA perform normal diffusion rather than sub-diffusion on lipid bilayers is because the oxDNA molecule used in this work is too short that the number of positively charged lipids attracted by the oxDNA is small. Hence, the energy well generated by such small number of cationic lipids is shallow from which oxDNA can easily escape. Moreover, the energy landscape that the 17 base pairs oxDNA experiences is not as heterogeneous as the long “bead-spring” chain experiences, as demonstrated by Figure 1. In addition, the temperature assigned to oxDNA beads is T=0.1, so the area that oxDNA traveled is smaller compared to that traveled by “bead-spring” chains.

Figure 10b shows relationship between the fitted 2D diffusion coefficients of both the middle and tail beads of oxDNA model and the temperature of lipids. As can be seen from Figure 10b, the temperature of lipid bilayer membranes has negligible effects on the diffusivity of oxDNA, keeping in mind that the temperature of oxDNA was fixed to 0.1 $k_{B}T/\epsilon$. Therefore, the simulation results of oxDNA on Cooke’s lipid bilayers at various temperatures presented here reflect the diffusive properties of oxDNA on heterogeneous surfaces with different rearranging rates of energy traps. However, since 17 base pairs oxDNA molecule is quite short as discussed above and travel distances of oxDNA are limited during our simulations, the effects of lipids lateral diffusion on oxDNA diffusivity can be neglected. Thus, to better understand the interactions between oxDNA and lipid bilayers, a different realization of oxDNA in LAMMPS package is required. By comparing the 2D diffusion coefficients of oxDNA bead in the middle of the chain to that of oxDNA bead at the end of the chain, it can be concluded that the motions of tail bead is faster due to less constraints, which is similar to the simulation results of “bead-spring” chains.

## 4. Conclusions

Using Brownian dynamics simulations, the diffusive behaviors of “bead-spring” chains on lipid bilayer membranes with various molar fractions of charged lipids have been investigated. Our simulation results of two-dimensional mean squared displacements show that DNA segments perform sub-diffusion on lipid membranes with exponent α≈0.38, which agrees with experimental results at short time scale [14]. The analysis of 1D displacement distributions of DNA beads demonstrates that non-Gaussian diffusive behaviors of DNA beads are absent for this simple model of DNA-lipid bilayers although they exhibit anomalous diffusion on membranes. This confirms the fact that viscoelasticity of medium as well as metabolic activities in cytoplasm are very critical for the non-Gaussian behaviors of cytoplasmic particles and loci. Moreover, our preliminary results of combining oxDNA coarse-grained model with Cooke’s generic lipid model show that a short double stranded DNA segment performs normal diffusion rather than sub-diffusion on lipid bilayer membranes. In addition, our simulation results indicate that the change of the rearranging rate of charged lipids within membranes has negligible effects on the diffusive motions of short oxDNA. However, to develop a mesoscopic model of DNA-lipid complexes based on oxDNA model and Cooke’s lipid model as a faster alternative of widely used coarse-grained model such as Martini FF, some modifications of oxDNA implementation in LAMMPS package have to be carried out so as to ensure that this mesoscopic model has precise predictions of DNA-lipid bilayer membranes interactions. Recently, Jarin et al. [59] investigated the interaction between Cooke’s lipids with peptide using Martini 3 model. Then, introducing oxDNA model into such a combination of lipids and peptide (or even protein) models would allow us to develop a new mesoscopic model which contains all the most important constituents of the cell.

## Data Availability

The data presented in this study are openly available in Mendeley Data at http://doi.org/10.17632/332hr7t7wb.1 (accessed on 22 February 2023).
